# Clinical impact of amyloid PET using ^18^F-florbetapir in patients with cognitive impairment and suspected Alzheimer’s disease: a multicenter study

**DOI:** 10.1007/s12149-022-01792-y

**Published:** 2022-10-04

**Authors:** Hiroshi Matsuda, Kyoji Okita, Yumiko Motoi, Toshiki Mizuno, Manabu Ikeda, Nobuo Sanjo, Koji Murakami, Taiki Kambe, Toshiki Takayama, Kei Yamada, Takashi Suehiro, Keiko Matsunaga, Takanori Yokota, Ukihide Tateishi, Yoko Shigemoto, Yukio Kimura, Emiko Chiba, Takahiro Kawashima, Yui Tomo, Hisateru Tachimori, Yuichi Kimura, Noriko Sato

**Affiliations:** 1 Department of Radiology, National Center of Neurology and Psychiatry, 4-1-1, Ogawa-higashi, Kodaira, Tokyo 187-8551 Japan; 2 Department of Biofunctional Imaging, Fukushima Medical University, 1 Hikariga-oka, Fukushima City, Fukushima 960-1295 Japan; 3 Drug Discovery and Cyclotron Research Center, Southern Tohoku Research Institute for Neuroscience, 7-61-2 Yatsuyamada, Koriyama, Fukushima 963-8052 Japan; 4 Integrative Bain Imaging Center, National Center of Neurology and Psychiatry, 4-1-1, Ogawa-higashi, Kodaira, Tokyo 187-8551 Japan; 5 Department of Diagnosis, Prevention, and Treatment of Dementia, Juntendo University Graduate School of Medicine, 3-1-3 Hongo, Bunkyo-ku, Tokyo 113-8431 Japan; 6 Department of Neurology, Kyoto Prefectural University of Medicine, 465 Kajiicho, Kamigyo Ward, Kyoto 602-8566 Japan; 7 Department of Psychiatry, Osaka University Graduate School of Medicine, 2-2 Yamadaoka, Suita, Osaka 565-0871 Japan; 8 Department of Neurology and Neurological Science, Tokyo Medical and Dental University Graduate School of Medical and Dental Sciences, 1-5-45 Yushima, Bunkyo-ku, Tokyo 113-8510 Japan; 9 Department of Radiology, Juntendo University Graduate School of Medicine, 3-1-3 Hongo, Bunkyo-ku, Tokyo 113-8431 Japan; 10 Department of Neurology, Juntendo University Graduate School of Medicine, 3-1-3 Hongo, Bunkyo-ku, Tokyo 113-8431 Japan; 11 Department of Psychiatry, Juntendo University Graduate School of Medicine, 3-1-3 Hongo, Bunkyo-ku, Tokyo 113-8431 Japan; 12 Department of Radiology, Kyoto Prefectural University of Medicine, 465 Kajiicho, Kamigyo Ward, Kyoto, 602-8566 Japan; 13 Department of Molecular Imaging in Medicine, Osaka University Graduate School of Medicine, 2-2 Yamadaoka, Suita, Osaka 565-0871 Japan; 14 Department of Diagnostic Radiology, Tokyo Medical and Dental University Graduate School of Medical and Dental Sciences, 2-2 Yamadaoka, Suita, Osaka 565-0871 Japan; 15 Department of Clinical Data Science, Clinical Research & Education Promotion Division, National Center of Neurology and Psychiatry, 4-1-1, Ogawa-higashi, Kodaira, Tokyo, 187-8551 Japan; 16 Faculty of Informatics, Cyber Informatics Research Institute, Kindai University、3-4-1, Kowakae, Higashiosaka, Osaka 577-8502 Japan

**Keywords:** Alzheimer’s disease, Amyloid, PET, ^18^F-florbetapir

## Abstract

**Objective:**

Amyloid positron emission tomography (PET) can reliably detect senile plaques and fluorinated ligands are approved for clinical use. However, the clinical impact of amyloid PET imaging is still under investigation. The aim of this study was to evaluate the diagnostic impact and clinical utility in patient management of amyloid PET using ^18^F-florbetapir in patients with cognitive impairment and suspected Alzheimer’s disease (AD). We also aimed to determine the cutoffs for amyloid positivity for quantitative measures by investigating the agreement between quantitative and visual assessments.

**Methods:**

Ninety-nine patients suspected of having AD underwent ^18^F-florbetapir PET at five institutions. Site-specialized physicians provided a diagnosis of AD or non-AD with a percentage estimate of their confidence and their plan for patient management in terms of medication, prescription dosage, additional diagnostic tests, and care planning both before and after receiving the amyloid imaging results. A PET image for each patient was visually assessed and dichotomously rated as either amyloid-positive or amyloid-negative by four board-certified nuclear medicine physicians. The PET images were also quantitatively analyzed using the standardized uptake value ratio (SUVR) and Centiloid (CL) scale.

**Results:**

Visual interpretation obtained 48 positive and 51 negative PET scans. The amyloid PET results changed the AD and non-AD diagnosis in 39 of 99 patients (39.3%). The change rates of 26 of the 54 patients (48.1%) with a pre-scan AD diagnosis were significantly higher than those of 13 of the 45 patients with a pre-scan non-AD diagnosis (*χ*^2^ = 5.334, *p* = 0.0209). Amyloid PET results also resulted in at least one change to the patient management plan in 42 patients (42%), mainly medication (20 patients, 20%) and care planning (25 patients, 25%). Receiver-operating characteristic analysis determined the best agreement of the quantitative assessments and visual interpretation of PET scans to have an area under the curve of 0.993 at an SUVR of 1.19 and CL of 25.9.

**Conclusion:**

Amyloid PET using ^18^F-florbetapir PET had a substantial clinical impact on AD and non-AD diagnosis and on patient management by enhancing diagnostic confidence. In addition, the quantitative measures may improve the visual interpretation of amyloid positivity.

## Introduction

Amyloid positron emission tomography (PET) can reliably detect senile plaques comprising amyloid β peptides, one of the hallmarks of Alzheimer’s disease (AD), and fluorinated ligands are approved for clinical use in several countries. In Japan, one of the approved tracers for amyloid PET imaging is ^18^F-fluorobetapir [1, 2]. This radiopharmaceutical, delivered as a final product to a clinical facility, has proven efficacy in the visualization of amyloid β plaques in the brains of patients with cognitive impairment and suspected AD (https://www.info.pmda.go.jp/go/pack/43004A2A1029_2_01/). However, the diagnostic impact and clinical utility of amyloid PET imaging are still under investigation. Although negative findings on amyloid PET can almost entirely rule out AD [3], the prevalences of positive scans in one meta-analysis were 88% in patients with AD, 51% in patients with dementia with Lewy bodies, 30% in patients with cerebrovascular disease, 12% in patients with frontotemporal dementia, 38% in patients with corticobasal degeneration, and 24% in healthy elderly individuals serving as controls [4]. In contrast, in other meta-analyses of the clinical impact of amyloid PET, the overall rates of changes in diagnosis and patient management after amyloid PET varied widely from 19 to 55% [5–13] and from 37 to 87% [8, 9, 11–16], respectively, from study to study. Consequently, due to the lack of definitive evidence supporting its clinical impact, amyloid PET is still not reimbursed by the Japanese health insurance system.

Visual interpretation is utilized to determine qualitatively if amyloid PET is positive or negative when it is employed in clinical practice. Equivocal results are unavoidable in this binary classification and cause inter-rater variability in visual interpretation [17]. The addition of quantitative analysis to visual interpretation has thus been suggested [18]. The standardized uptake value ratio (SUVR) has been extensively employed in the quantitative study of amyloid PET. Additionally, the Centiloid (CL) scale has recently been adopted [19, 20], which harmonizes the quantitative amyloid imaging measurements by standardizing the results of each analytical method or PET ligand.

The aim of this multicenter study was to evaluate the diagnostic impact and clinical utility in patient management of amyloid PET using ^18^F-florbetapir in patients with cognitive impairment and suspected AD. We also aimed to determine the cutoffs for amyloid positivity for the SUVR and CL in quantitative analysis by investigating the agreement between the quantitative and visual assessments of ^18^F-florbetapir PET.

## Materials and methods

### Participants

In total, 103 Japanese patients (56 women and 47 men; range, 43–88 years) were recruited from five participating centers with a specialized unit for dementia (Table 1). General cognition was assessed using the Mini-Mental State Examination (MMSE) [21]. Inclusion criteria were as follows: a 15–85% diagnostic confidence that the cognitive impairment is due to Alzheimer’s disease based on clinical criteria for AD according to the National Institute on Aging and the Alzheimer’s Association [22] or for neurocognitive disorder according to the Diagnostic and Statistical Manual of Mental Disorder-V [23] before amyloid PET and brain magnetic resonance imaging (MRI; T1-weighted, T2-weighted, and FLAIR imaging) conducted up to 90 days before patient registration. Exclusion criteria were as follows: no cognitive decline; the presence of gross lesions, such as a brain tumor, cerebrovascular malformation, or cortical infarction on MRI; and advanced dementia with a MMSE score below 19. Four patients who passed the screening withdrew consent before the PET scan. Finally, 99 patients (47 men and 52 women; range, 43–88 years) were included in this study.

**Table 1:** Details of the PET imaging and image reconstruction methods in each center

PET study institute		Tokyo 1 (Kodaira)	Kyoto	Tokyo 2 (Bunkyo-ku)	Osaka	Tokyo 3 (Bunkyo-ku)
Number of subjects studied		50 (30 Patients and 20 young healthy controls)	15 Patients	26 Patients	15 patients	13 patients
PET imaging	Scanner	Siemens biograph 16 truepoint	Siemens biograph horizon-4R	Siemens biograph mCT flow	Shimadzu Eminence SOPHIA SET-3000 BCT/X	Canon Celesteion
	Detector	Lu2SiO5	Lu2SiO5	Lu2SiO5	Bismuth germanate	lutetium-based scintillator
	Attenuation correction	CT	CT	CT	137Cs	CT
	Injection dose (MBq)	390 ± 10	359 ± 31	375 ± 18	363 ± 19	370 ± 0
	Start time (min)	40.2 ± 1.5	40.4 ± 1.2	40.0 ± 0.0	40.0 ± 0.0	40.0 ± 0.0
	Scan time (min)	20	20	20	20	20
	Acquisition mode	List mode	List mode	List mode	List mode	List mode
Image reconstruction	Correction for scatter counting	Single scatter simulation	Single scatter simulation	Single scatter simulation	Deconvolution method	Single scatter Simulation
	Correction for random coincidence counting	Delayed coincidence correction	Delayed coincidence correction	Delayed coincidence correction	Delayed coincidence correction	Delayed coincidence correction
	Image reconstruction	3D-OSEM	TrueX + TOF	High definition PET	2D-OSEM	3D-OSEM
	iteration/subset	4/14	10/10	4/21	4/16	4/20
	Post filter	–	–	Gaussian 4 mm FWHM	Gaussian 5 mm FWHM	–
	Voxel Size	2.0 mm (X) × 2.0 mm(Y) × 2.03 mm(Z)	0.72 mm(X) × 0.72 mm(Y) × 2.0 mm (Z)	2.04 mm(X) × 2.04 mm(Y) × 2.0 mm(Z)	2.0 mm(X) × 2.0 mm(Y) × 3.25 mm(Z)	2.0 mm(X) × 2.0 mm(Y) × 2.0 mm(Z)
	Matrix Size	168 pixels × 168 pixels × 81 slices	512 pixels × 512 pixels × 111 slices	200 pixels × 200 pixels × 111 slices	128 pixels × 128 pixels × 79 slices	160 pixels × 160 pixels × 96 slices

*CT* computed Tomography, *OSEM* ordered subsets expectation maximization, *FWHM* full width at half maximum

In addition, 22 Japanese cognitively normal healthy subjects (13 men and 9 women; range, 35–50 years old) were recruited from one participating center for the purpose of establishing an amyloid-negative database. Inclusion criteria were as follows: Japanese individuals between the ages of 35 and 50 years and an MMSE of 29 or higher without any medical history of neuropsychiatric disease. Two subjects were excluded during screening due to a MMSE score below 29. Finally, 20 subjects (13 men and 7 women; range, 35–50 years) were included in this study.

### Clinical protocol

Site-specialized physicians for dementia provided a diagnosis of AD or non-AD with a percentage estimate of their confidence and their plan for patient management in terms of medication, prescription dosage, additional diagnostic tests, and care planning both before and after receiving the results from amyloid imaging with ^18^F-florbetapir. Non-AD diagnosis included mild cognitive impairment (MCI), dementia with Lewy bodies, vascular dementia, frontotemporal dementia, depression, idiopathic normal pressure hydrocephalus, progressive supranuclear palsy, corticobasal degeneration, and epilepsy. The diagnoses with the highest percentage of assigned confidence were regarded as the pre-scan and post-scan diagnoses.

### PET imaging

Each PET imaging site, together with the PET camera, satisfied the image quality criteria defined by the Japanese Society of Nuclear Medicine in which a Hoffman 3D brain phantom and a uniform cylindrical phantom are applied (http://jsnm.org/wp_jsnm/wp-content/themes/theme_jsnm/doc/StandardPETProtocolPhantom20170201.pdf) [24]. ^18^F-florbetapir was intravenously injected as a slow bolus in an antecubital vein at a mean ± standard deviation (SD) dose of 377 ± 20 MBq (range, 293–422 MBq). A 20-min list-mode PET scan was started from 40.1 ± 1.0 min (range, 39–44 min) according to the imaging acquisition guidelines of the Amyvid® package insert (https://www.accessdata.fda.gov/drugsatfda_docs/label/2012/202008s000lbl.pdf), which recommends that the PET scan starts 30–50 min after Amyvid® injection. In all participating institutions, all appropriate corrections, including scatter and time-of-flight, were applied with a low-dose computed tomography scan or radioactive source (^137^Cs) for attenuation correction (Table 1). Images were reconstructed using the ordered subset expectation maximization (OSEM) method. Clinical status was checked before and after PET scanning in each participant. Subjects were observed for adverse events from the administration of tracer and immediately after the PET scan.

### Visual interpretation and quantitative image analysis

A static 20-min PET image 40–60-min post-injection from each patient was visually assessed and dichotomously rated as either amyloid-positive or amyloid-negative by four board-certified nuclear medicine physicians (H.M., Y.S., Yuk.K., and E.C.). All physicians had completed the electronic training program (https://amyvid-training.pdradiopharma.com/login/) developed by PDRadiopharma Inc. for the interpretation of ^18^F-florbetapir images and were certified by the Japanese Society of Nuclear Medicine after passing a subsequent visual interpretation training program. The four readers were blinded to clinical information and independently interpreted the PET images according to the training program instructions. The review included all transaxial slices of the brain using a black and white scale with the maximum intensity of the scale set to the maximum intensity of all brain voxels. Negative scans show more radioactivity in white matter than in gray matter, creating a clear gray–white contrast. In contrast, positive scans show cortical areas with reduction or loss of the normally distinct gray–white contrast. These scans have one or more areas with increased cortical gray matter signal, which results in reduced or absent gray–white contrast. Specifically, a positive scan will have either: a) two or more brain areas (each larger than a single cortical gyrus) in which there is reduced or absent gray–white contrast, or b) one or more areas in which gray matter radioactivity is intense and clearly exceeds radioactivity in adjacent white matter. The four readers shared their results. In cases where the four readers reached different conclusions, the conclusion reached by the highest number of readers was adopted. If two pairs of readers each reached different conclusions, the visual rating was rerun until the readers reached consensus for each case.

Quantitative analysis of ^18^F-florbetapir PET was performed using our software developed in-house Amyquant^®^ [25] with a SUVR and a 100-point CL scale. The CL scale assigns an average value of zero in high-certainty amyloid-negative subjects and an average of 100 in typical AD patients. In the processing pipeline, first, the individual MRI was reoriented and coregistered to the Montreal Neurological Institute (MNI) template (avg152T1.nii) provided with Statistical Parametric Mapping 12 software (https://www.fil.ion.ucl.ac.uk/spm). The individual PET was reoriented and coregistered to the coregistered individual MRI. Then, the coregistered individual MRI was warped into MNI space using unified segmentation in SPM12. The parameters of the deformation field in this warping were applied to the coregistered individual PET for anatomic standardization into MNI space. The SUVR was calculated from ^18^F-florbetapir PET counts in the global cortical target region and in the whole cerebellum as a reference region using CL standard volumes of interest (http://www.gaain.org/centiloid-project). Then, the SUVR was converted to CL values using a direct conversion equation (CL = 175.17 × SUVR − 182.23), as described in a previous report [19]. We calculated the SUVR_40–50_, SUVR_50–60_, and SUVR_40–60_, as well as the CL_40–50_, CL_50–60_, and CL_40–60_, from PET images obtained 40–50-min, 50–60-min, and 40–60-min post-injection, respectively.

### Endpoints

The primary endpoint of the study was a change in diagnosis from AD to non-AD and vice versa between pre- and post-amyloid PET scans as well as associated changes in patient management in terms of medication, prescription dosage, additional diagnostic tests, and care planning. The secondary endpoint was the determination of cutoffs for quantitative assessments that showed the best agreement with positive or negative results obtained via the visual interpretation of ^18^F-florbetapir PET images.

### Statistical analysis

Mean ± SD values and frequency distributions are reported. Differences between groups were tested using a Welch’s *t* test or analysis of variance, Tukey–Kramer Honest Significant Difference test, and Pearson χ^2^ tests when appropriate. Concordances of SUVR and CL scales between PET images obtained 40–60 min, 40–50 min, and 50–60 min were assessed using Bland–Altman plots. Optimal cut-off values for the SUVR and CL in quantitative assessment showing the best agreement with visual interpretation were determined using Youden’s index (YI) and the maximal accuracy calculated from receiver-operating characteristic (ROC) analysis. The cut-point derived by YI optimizes the ability of a test to differentiate when equal weight is given to sensitivity and specificity. It is defined mathematically as: YI = sensitivity + specificity—1 [26]. Agreement between visual and quantitative assessments of the ^18^F-florbetapir classification, as well as the inter-rater agreement for visual interpretations, was assessed using Cohen’s kappa. All statistical tests were performed using JMP ver. 16.2.0 (SAS Institute) and R^®^ ver.3·5 or later (R Foundation for Statistical Computing).

## Results

No adverse events were observed after the administration of the tracers or immediately after the PET scan in all subjects.

The mean ± SD MMSE score of the 99 patients was 24.6 ± 3.2 (range 20–30). No significant differences were found in demographic characteristics between the 54 patients with a pre-scan AD diagnosis and the 45 patients with a pre-scan non-AD diagnosis (Table 2). The patients with a pre-scan AD diagnosis showed significantly lower MMSE scores compared with those with a pre-scan non-AD diagnosis (*p* < 0.001).

**Table 2:** Demographics and clinical characteristics of patients based on pre-scan diagnosis

	Pre-scan diagnosis		*p* Value
	AD (*n* = 54)	Non-AD (*n* = 45)	
Demographic characteristics			
Age, years, mean ± SD	72.3 ± 10.2	72.6 ± 10.1	0.8715
Sex, female/male, n	31/23	21/24	0.3828
Education, years, mean ± SD	13.2 ± 3.1	13.7 ± 2.9	0.4028
Neuropsychological evaluation			
Mini-mental state examination, mean ± SD	23.5 ± 2.9	26.0 ± 3.0	< 0.001

**Fig. 1:**
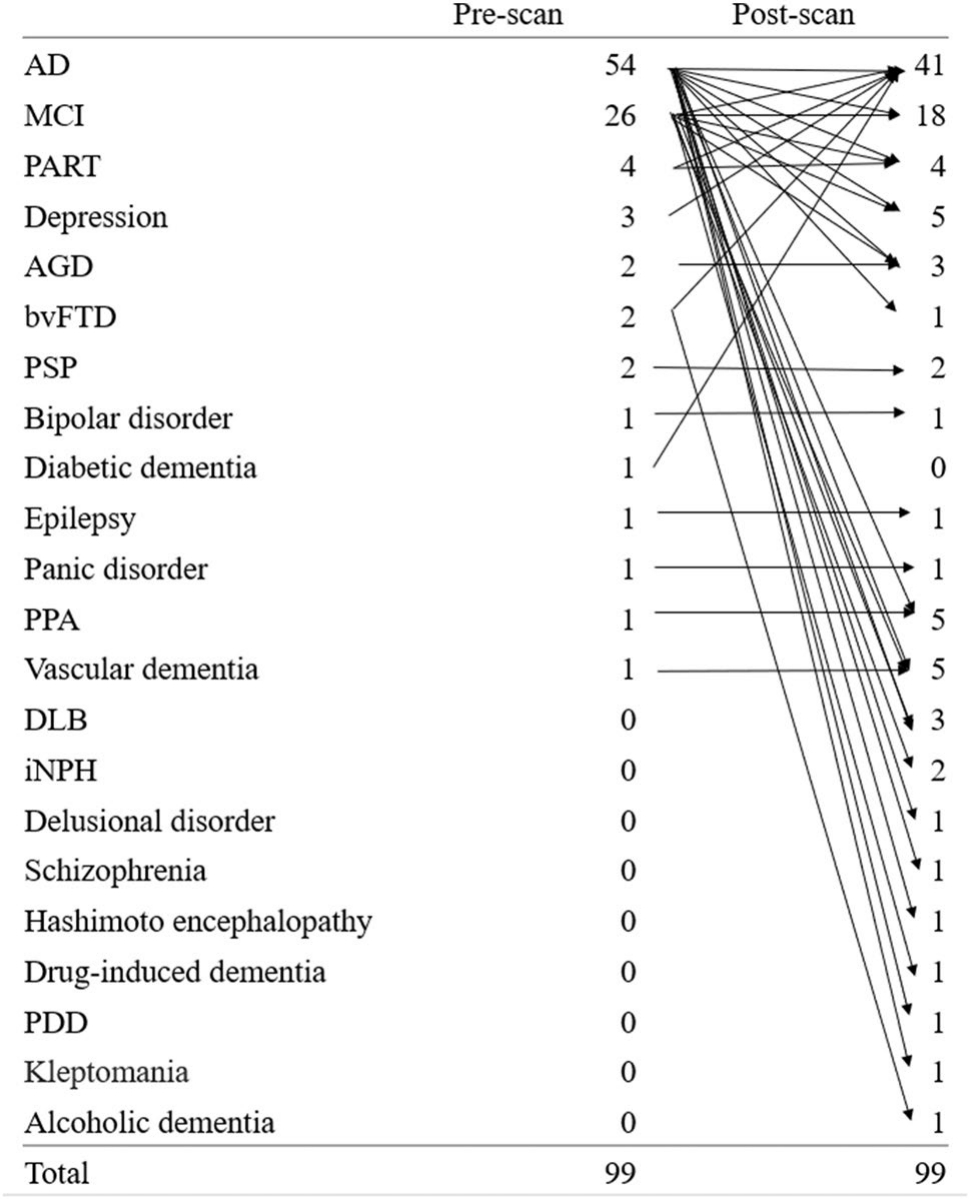
Details of diagnostic changes at the pre- and post-amyloid PET scan. *AD* Alzheimer’s disease, *MCI* Mild cognitive impairment, *PART* Primary age-related tauopathy, *AGD* Argyrophilic grain disease, *bvFTD* behavioral variant frontotemporal dementia, *PSP* Progressive supranuclear palsy, *PPA* Primary progressive aphasia, *DLB* Dementia with Lewy bodies, *iNPH* idiopathic normal pressure hydrocephalus, *PDD* Pervasive developmental disorders

Visual interpretation obtained 48 positive and 51 negative PET scans. The prevalence of amyloid-positive scans was not significantly different (*χ*^2^ = 0.539, *p* = 0.462) between patients with a pre-scan AD diagnosis (29 of 54, 53.7%) and those with a non-AD diagnosis (19 of 45, 42.2%). Amyloid PET results led to changes in the AD and non-AD diagnoses in 39 of 99 patients (39.3%), with significantly higher rates in patients with pre-scan AD (26 of 54, 48.1%) than in patients with non-AD diagnosis (13 of 45, 28.8%) (*χ*^2^ = 5.334, *p* = 0.0209). Details of the pre-scan and post-scan diagnostic changes are shown in Fig. 1. The diagnostic confidence of AD significantly increased for patients with an unchanged diagnosis of AD (Δ = 22.7% ± 13.2%, *p* < 0.0001) and for patients whose diagnosis changed from non-AD to AD (Δ = 46.1% ± 19.4%, *p* < 0.0001) after the disclosure of the amyloid PET results. Meanwhile, the diagnostic confidence of AD significantly decreased in patients whose diagnosis changed from AD to non-AD (Δ =  − 53.2% ± 13.5%, *p* < 0.0001) and in patients with an unchanged diagnosis of non-AD (Δ =  − 13.2% ± 30.2%, *p* = 0.0094) after amyloid PET (Table 3). Overall, 41 of the 48 patients (85.4%) with amyloid-positive results received a post-scan diagnosis of AD, and the remaining seven patients with amyloid-positive results received a post-scan diagnosis of MCI in six patients and primary progressive aphasia in one patient. All of the 51 patients with amyloid-negative results received a post-scan diagnosis of non-AD.

**Table 3:** Changes of diagnostic confidence of AD (%) before and after amyloid PET

Diagnosis	Amyloid PET		Diagnostic confidence of AD (%)			*p* Value
Pre-scan → Post-scan	Positive	Negative	Pre-scan	Post-scan	Δ	
AD → AD	28	0	66.1 ± 12.0	88.8 ± 9.1	22.7 ± 13.2	< 0.0001
AD → non-AD	0	26	60.4 ± 11.0	7.1 ± 14.1	− 53.2 ± 13.5	< 0.0001
Non-AD → non-AD	7	25	28.4 ± 9.4	15.1 ± 26.9	− 13.2 ± 30.2	0.0094
Non-AD → AD	13	0	31.2 ± 10.8	77.3 ± 14.1	46.1 ± 19.4	< 0.0001

Amyloid PET results led to at least one change in the patient management plan in 42 of 99 patients (42%), without significant differences between patients with pre-scan AD and those with non-AD diagnosis (*χ*^2^ = 0.001, *p* = 0.970, Table 4). Amyloid PET results thereafter led to changes in medication, prescription dosage, additional diagnostic tests, and care planning in 20 (20%), 5 (5%), 4 (4%), and 25 (25%) patients, without significant differences in medication (*χ*^2^ = 0.301, *p* = 0.583), additional diagnostic tests (*χ*^2^ = 3.474, *p* = 0.0624), and care planning (*χ*^2^ = 2.854, *p* = 0.091) but with a significant difference in prescription dosage (*χ*^2^ = 4.388, *p* = 0.0362) between patients with pre-scan AD and those with a non-AD diagnosis.

**Table 4:** Changes of patient management in pre-scan AD and non-AD diagnosis after amyloid PET

	Change status	Pre-scan diagnosis		
		AD	Non-AD	Total
Overall patient management	Unchanged	31	26	57
	Changed	23	19	42
Medication	Unchanged	42	37	79
	Changed	12	8	20
Prescription dosage	Unchanged	49	45	94
	Changed	5	0	5
Additional diagnostic tests	Unchanged	50	45	95
	Changed	4	0	4
Care planning	Unchanged	44	30	74
	Changed	10	15	25

**Fig. 2:**
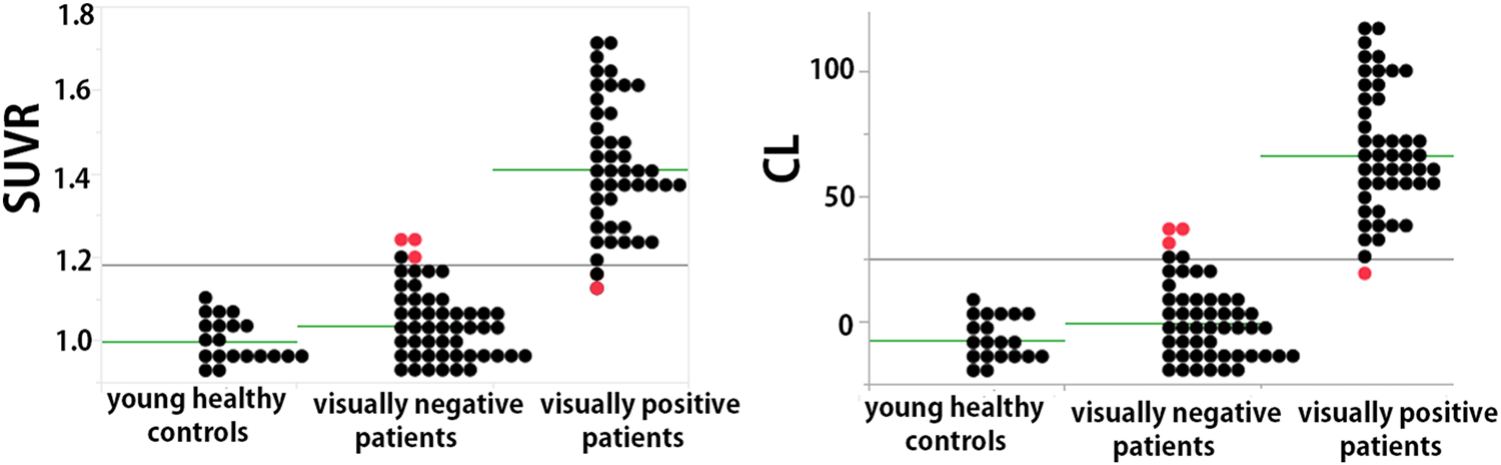
SUVR_40–60_ and CL_40–60_ values in scans of young healthy controls and in visually amyloid-negative and amyloid-positive patients. Significant differences in the SUVR_40–60_ and CL_40–60_ scales were observed between visually positive scans and visually negative scans and between visually positive scans and scans of young healthy controls (*p* < 0.0001). There were no significant differences in these values between scans of young healthy controls and visually negative scans (*p* > 0.05). Discordant patients between quantitative measures and visual interpretation are marked using red symbols

In a visual interpretation of the positive or negative findings of amyloid PET, four readers completely agreed in 78 of 99 scans of the patients (79%). The Cohen’s kappa agreement between two of each of the four readers ranged from 0.718 to 0.778 (0.743 ± 0.026). SUVR_40–60_ values were 1.00 ± 0.05, 1.04 ± 0.08, and 1.41 ± 0.15 for scans of young healthy controls, visually negative scans, and visually positive scans, respectively, whereas CL_40_60_ scales were − 7.6 ± 8.4, − 0.7 ± 15.3, and 66.2 ± 24.9, respectively (Fig. 2). Significant differences in the SUVR_40–60_ and CL_40–60_ scales were observed between visually positive scans and visually negative scans and between visually positive scans and the scans of young healthy controls (*p* < 0.0001). There were no significant differences in these values between the scans of young healthy controls and visually negative scans (*p* > 0.05).

ROC analysis determined the best agreement of quantitative assessments and visual interpretation of ^18^F-florbetapir PET scans obtained 40–60-min post-injection to have an area under the curve of 0.993 at an SUVR of 1.19 and CL of 25.9. If visual interpretation was considered the standard of truth, quantitative assessment demonstrated 97.9% sensitivity, 94.1% specificity, and 95.9% accuracy. Using these cut-off values, there was strong agreement between them (Cohen’s kappa = 0.92). The SUVR and CL values of the four discordant cases between quantitative assessments and visual interpretation ranged from 1.15 to 1.24 and from 19.2 to 35.3, respectively. These discordant cases were classified into two patterns. Diffuse elevation of cortical activity was regarded as visually amyloid-negative despite a relatively high SUVR or CL in three cases. In contrast, mildly focal elevation of cortical activity in two areas was regarded as visually amyloid-positive despite a relatively low SUVR or CL in one case.

**Fig. 3:**
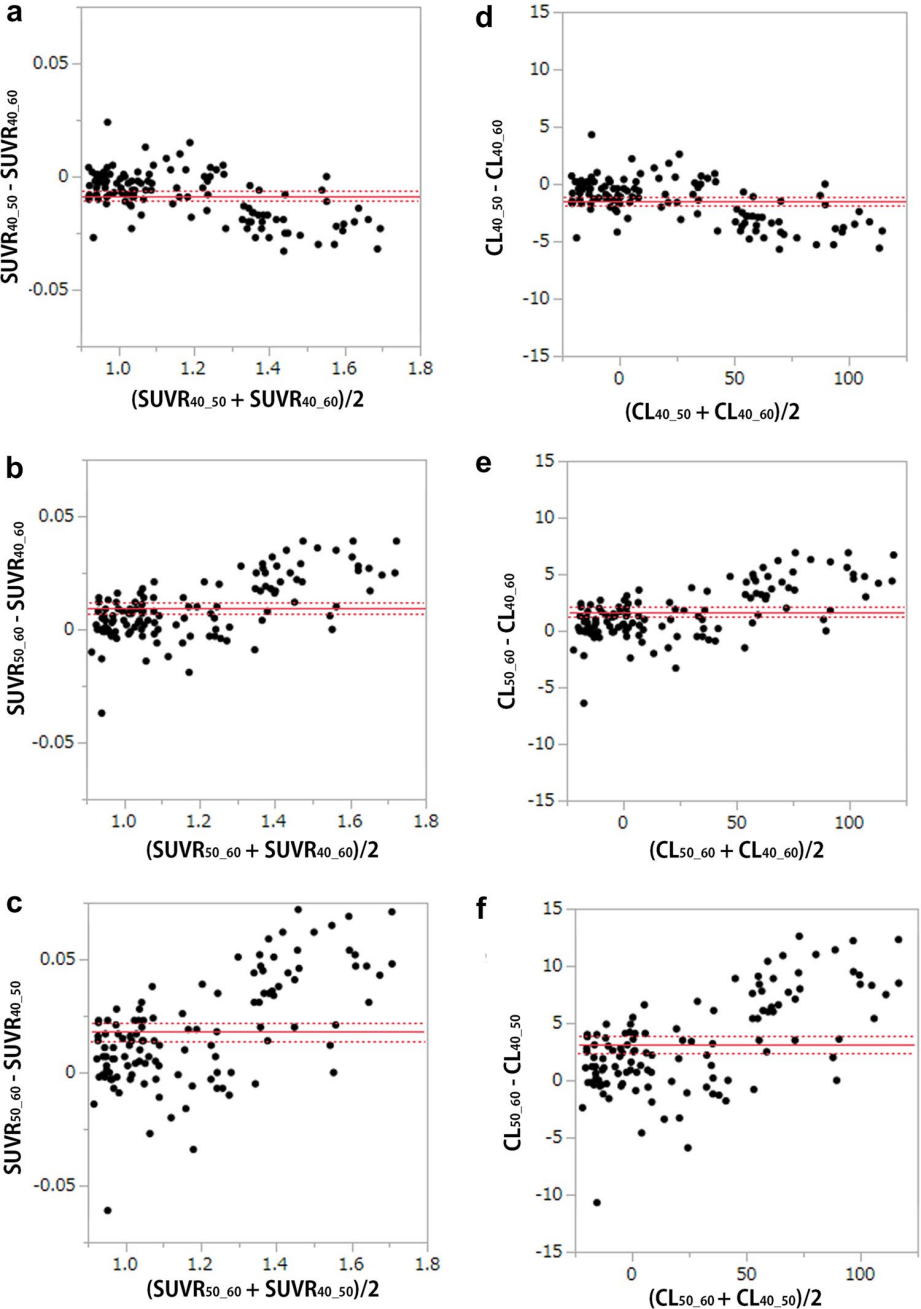
Comparison of the SUVR and CL values among different start time and imaging time conditions. In the Bland–Altman plot, there were significant differences among the SUVR_40_50_, SUVR_50_60_, and SUVR_40_60_ and among the CL_40–50_, CL_50–60_, and CL_40–60_ scales (*p* < 0.001), while Spearman correlation analysis identified significant associations (all *p* < 0.001) between the difference in the SUVR_40_50_ versus SUVR_40_60_ and SUVR load (**a**, *ρ* =  − 0.514), between the difference in the SUVR_50–60_ versus SUVR_40–60_ and SUVR load (**b**, *ρ* = 0.568), between the difference in the SUVR_50–60_ versus SUVR_40–50_ and SUVR load (**c**, *ρ* = 0.549), between the difference in the CL_40–50_ versus CL_40–60_ and CL load (**d**, *ρ* =  − 0.513), between the difference in the CL_50–60_ versus CL_40–60_ and CL load (**e**, *ρ* = 0.572), and between the difference in the CL_50–60_ versus CL_40–50_ and CL load (**f**, *ρ* = 0.541)

**Fig. 4:**
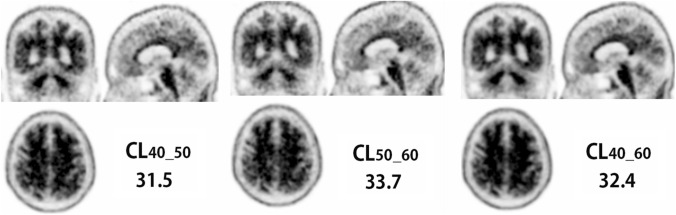
Representative PET images obtained 40–50 min, 50–60 min, and 40–60 min post-injection in a patient with pre-scan AD diagnosis. CL_50–60_ is slightly higher than CL_40–50_; CL_40–60_ is in between

The SUVR_40–50_, SUVR_50–60_, and SUVR_40–60_ of all 99 patients and 20 young healthy controls were 1.17 ± 0.22, 1.19 ± 0.23, and 1.18 ± 0.22, respectively, while the CL_40–50_, CL_50–60_, and CL_40–60_ scales of all subjects were 23.6 ± 37.8, 26.7 ± 40.4, and 25.1 ± 38.9, respectively. In a Bland–Altman plot, there were significant differences among the SUVR_40–50_, SUVR_50–60_, and SUVR_40–60_ and among the CL_40–50_, CL_50–60_, and CL_40–60_ scales, while Spearman correlation analysis identified a significant association between the difference among the SUVR_40–50_, SUVR_50–60_, and SUVR_40–60_ and SUVR load and among the CL_40–50_, CL_50–60_, and CL_40–60_ scales and CL load (all *p* < 0.001, Fig. 3a–f). Representative PET images obtained 40–50 min, 50–60 min, and 40–60 min post-injection are shown in Fig. 4 along with their respective CL scales.

## Discussion

The current multicenter study examined the clinical impact of amyloid PET on the diagnosis and management of cognitively impaired patients with probable AD and of those with possible AD, but other disease was more likely. This is the first report of a clinical impact study of amyloid PET using ^18^F-florbetapir in Japan. The details of the diagnosis changed by amyloid PET have never been reported before. Amyloid PET results changed the etiologic diagnosis of AD or non-AD in 39.3% of all patients. The change rates were significantly higher for pre-scan AD diagnosis (48.1%) than for pre-scan non-AD diagnosis (28.8%). These change rates are comparable to those in previous multicenter studies.

Grundman et al. [5] reported changes in diagnosis after disclosure of the PET results using ^18^F-florbetapir in 125 of 229 patients (54.6%) as well as in 37.2% of patients with a pre-scan AD diagnosis and 61.9% of those with a pre-scan non-AD diagnosis. Another multicenter study using ^18^F-florbetapir [11] demonstrated changes in diagnosis after disclosure of the PET results in 62 of 228 patients (27.2%) as well as in 27.9% of patients with a pre-scan AD diagnosis and 25.4% of those with a pre-scan non-AD diagnosis. In the large-scale IDEAS study [13], amyloid PET results led to changes in the etiologic diagnosis from AD to non-AD in 2869 of 11,409 patients (25.1%) and from non-AD to AD in 1201 of 11,409 patients (10.5%). This higher change rate from AD to non-AD diagnosis compared with that from non-AD to AD agreed well with the results of the present study. The higher rate of change in pre-scan AD may be because AD can be ruled out when amyloid PET is negative. Furthermore, focusing on the details of the diagnosis, while pre-scan AD and MCI diagnoses decreased after the amyloid PET, post-scan diagnoses became further subdivided, increasing from 13 different diagnoses to 21 different diagnoses. This subdivision suggests the contribution of amyloid PET to more confirmatory diagnoses. In contrast, pre-scan non-AD diagnosis of 13 patients (6 MCI, 2 primary age-related tauopathy, 3 depression, 1 bvFTD, and 1 diabetic dementia) was changed to post-scan AD diagnosis. The considerable amyloid positivity with 59.3 ± 25.9 of CL scales led to the elevation of diagnostic confidence of AD.

These diagnostic changes led to changes in management in 42% of the patients, mainly in medication and care planning. These change rates were also comparable to those from previous investigations. The most common reported change in patient management due to amyloid PET results is a change in medication, ranging from 20 to 60% of cases [8, 11, 12, 14, 16]. In two previous studies, the care plan was changed in 10.9% [8] and 46.4% [9] of cases, respectively. The amyloid PET results also led to changes in prescription dosage and additional diagnostic tests in a small number of patients with a pre-scan AD diagnosis, as demonstrated in previous reports [7, 14, 16]. Thus, amyloid PET had a considerable impact on change in diagnosis and patient management by improving diagnostic confidence.

The inter-rater agreement of visual interpretation for amyloid positivity using ^18^F-florbetapir was similar to that in previous reports, where κ ranged from 0.69 to 0.71 [18, 27]. The high agreement of 95.9% of quantitative measures with the final decision regarding the visual interpretation may indicate the usefulness of CL scales as an adjunct to visual interpretation. The optimal CL cut-off values for amyloid positivity have been published by numerous investigations. A cut-off of 12.2 CL detected moderate-to-frequent CERAD neuritic plaques, while a cut-off of 24.4 CL identified intermediate-to-high AD neuropathological changes, according to a multicenter study using ^11^C-PiB [28] that looked at the relationship between antemortem amyloid PET and standard postmortem measures of AD neuropathology. Similar research employing ^11^C-PiB or ^18^F-florbetaben [29] found that the optimal threshold for finding moderate-to-frequent CERAD neuritic plaques was 20.1 CL, while the best cut-off for excluding neuritic plaques was a CL of 10. A favorable visual interpretation showed good agreement with results over 26 CL, according to that study's report. As for ^18^F-florbetapir, Clark et al. [30] demonstrated that an SUVR cut-off of 1.10 distinguished negative and positive ^18^F-florbetapir uptake relative to autopsy comparison of sparse/none versus moderate/frequent amyloid plaques. Royse et al. [31] also reported a similar SUVR cut-off of 1.11. In a comparative study of ^11^C-PiB and ^18^F-florbetapir, Navitsky et al. [20] translated an SUVR threshold for amyloid positivity to 24.1 CL. This cut-off value is very close to the 25.9 obtained in the present study as a comparison with visual interpretation. It should be noted here, however, that the start time of 40 min post-injection and imaging time of 20 min for the PET images used in the present visual interpretation differed from those of the study by Navistky et al. [20], which started 50 min post-injection with a 10-min data acquisition. The present study demonstrated slight but significant CL changes depending on start time and imaging time. The CL_50_60_ value is approximately 8% higher than the CL_40–60_ value, and the difference is more prominent for higher CL values. Thus, when a CL_50–60_ value is applied to ROC analysis, the threshold for amyloid positivity slightly elevates to 27.7.

In the present study, quantitative values were also obtained from young healthy controls who were younger than 50 years of age, because the presence of amyloid β deposition in postmortem studies was found to be relatively rare before 50 years of age [32]. Although the SUVR and CL scales in this young control group were lower than those in visually amyloid-negative patients, the differences were not statistically significant. These amyloid-negative PET images from young healthy controls may be further applied to a software program for Z-score analysis [25] as a negative control database to localize the significant amyloid accumulation. In contrast with an AD-related increase in amyloid deposition in the posterior cingulate gyrus, precuneus, and frontal cortex, an age-related increase in amyloid deposits was specifically observed in the temporal neocortex [33].

This study has several limitations. First, the nonrandomized design and lack of a control group limit the direct attribution of changes in management to PET. Second, the observed changes in diagnosis and management represent the behavior of specialized physicians rather than an evidence-based standard of care. Third, because no postmortem data were available, the lack of a gold standard hampered our ability to relate the findings to the underlying neuropathology. Fourth, the sample size was not particularly large.

## Conclusion

The present multicenter study suggested that ^18^F-florbetapir PET can exert a considerable clinical impact on AD and non-AD diagnosis and on patient management, particularly for medication and care planning, by improving the diagnostic confidence of AD. CL scale measures may help in the visual interpretation of amyloid positivity.
